# Upper gastrointestinal tract microbiota with oral origin in relation to oesophageal squamous cell carcinoma

**DOI:** 10.1080/07853890.2023.2295401

**Published:** 2023-12-27

**Authors:** Shegan Gao, Zichao Zhang, Kui Sun, Meng-Xiang Li, Yi-Jun Qi

**Affiliations:** a State Key Laboratory of Esophageal Cancer Prevention and Treatment, Henan Key Laboratory of Microbiome and Esophageal Cancer Prevention and Treatment, Henan Key Laboratory of Cancer Epigenetics, Cancer Hospital, The First Affiliated Hospital, College of Clinical Medicine, Medical College of Henan University of Science and Technology, Luoyang, China; b Department of Mathematics and Physics, Luoyang Institute of Science and Technology, Luoyang, China

**Keywords:** Oesophageal squamous cell carcinoma, tooth loss, periodontitis, microbiota, *porphyromonas gingivalis*

## Abstract

**Introduction:** Poor oral hygiene is linked to high risks of many systemic diseases, including cancers. Oral dysbiosis is closely associated with poor oral hygiene, causing tooth loss, gingivitis, and periodontitis. We provide a summary of studies and discuss the risk factors for oesophageal squamous cell carcinoma (ESCC) from a microbial perspective in this review.**Methods:** A literature search of studies published before December 31, 2022 from PubMed, Web of Science, and The Cochrane Library was performed. The search strategies included the following keywords: (1) oral care, oral health, oral hygiene, dental health, dental hygiene, tooth loss, teeth loss, tooth absence, missing teeth, edentulism, tooth brushing, mouthwash, and tooth cleaning; (2) esophageal, esophagus, oesophagus, and oesophageal; (3) cancer, carcinoma, tumor, and neoplasm.**Discussion:** Poor oral health, indicated by infrequent tooth brushing, chronic periodontitis, and tooth loss, has been associated with an increased risk of squamous dysplasia and ESCC. Oral microbial diversity and composition are profoundly dysregulated during oesophageal tumorigenesis. Similar to the oral microbiota, the oesophageal microbiota varies distinctly in multiple bacterial taxa in ESCC and gastric cardia adenocarcinoma, both of which have high co-occurrence rates in the “Oesophageal Cancer Belt”. In addition, the potential roles of oncogenic viruses in ESCC have also been discussed. We also briefly explore the potential mechanisms underlying the tumor-promoting role of dysregulated microbiota for the development of therapeutic targeting strategies.**Conclusion:** Poor oral health is an established risk indicator of ESCC. The dysbiosis of microbiota in upper gastrointestinal tract that highly resembles the oral microbial ecosystem but with distinct features at individual sites contributes to the development and progression of ESCC.

## Introduction

Oesophageal cancer ranks seventh and sixth in terms of incidence and death of cancer, respectively [1]. Two predominant histologic subtypes of oesophageal cancer, oesophageal squamous cell carcinoma (ESCC) and oesophageal adenocarcinoma (EAC), share few features in aetiology, regional distribution pattern, and time trend with the exception of poor prognosis [2]. Although EAC incidence has increased several folds over the past 40 years, ESCC still accounts for about 90% of oesophageal cancer globally at present, half of which occur in China [3]. The aetiology of ESCC varies by population. In China, Central Asia, and Sub-Saharan Africa, the major risk factors for ESCC comprise micronutrient deficiency, dietary carcinogen exposure, hot food and beverage ingestion, and low socioeconomic status [4–7]. In contrast, tobacco and alcohol consumption are important risk factors for ESCC in Western populations [8, 9]. Recently, chronic periodontitis and their related oral microorganisms have been linked to increased risks of various systemic diseases [10–13], including total cancer and certain site-specific cancers like ESCC [14–16].

Epidemiological studies suggest a causal link between infection-driven chronic inflammation and cancer [17, 18]. In general, approximately 20% of human tumours are associated with infectious agents, including virus, bacteria, and parasites [19]. Chronic inflammation is considered as the main risk factor for many cancers and contributes to acquisition of the majority of core cancer hallmarks [18]. The inflammatory milieu gives rise to large amounts of cytokines and growth factors, which foster the initiation and development of malignancies *via* accumulation of genetic instability, mutations, and epigenetic abnormalities [20–23]. Although one or several microbes can cause specific infection, the notion that the entire microbial community, termed microbiota, is reputed to be a pathogen, is becoming increasingly accepted [24, 25]. The human microbiota co-exists with its host in symbiosis and is beneficial for many physiological processes, such as nutrition absorption, metabolism, mucosal immune system development, and immune modulation [26–28]. Under conditions of dysbiosis, the dysregulated microbial community can promote the formation and development of many diseases including cancer, in synergism with other risk factors (e.g. smoking, alcohol intake, and dietary carcinogens).

Poor oral health, generally reflected by the degree of tooth loss, caries, and periodontitis, has been linked to a high risk of ESCC [15, 29–35]. Increasing evidence shows that health state of oral cavity is associated with oral microbiota, which has been implicated in the pathogenesis and development of various diseases [36]. The microbiota of the upper gastrointestinal tract has attracted less attention, particularly in relation to ESCC, compared with the microflora in the lower gastrointestinal tract. This review provides a summary of studies that have evaluated the effects of tooth loss, periodontitis, oral health, and practices of oral hygiene on the initiation and development of ESCC. In the light of culture-based and recent sequencing-based microbial profiling studies, we elaborate on latest data on microbiota in the upper gastrointestinal tract in relation to normal oesophageal mucosae and ESCC and assess the impact of microbial phylotypes on the carcinogenesis of ESCC. In addition, we summarize the associations between some extensively studied oncogenic viruses and ESCC. The potential tumorigenic mechanisms of microbiota dysbiosis in the context of ESCC are discussed.

## Oral milieu

Several epidemiological studies support the association between an increased risk of ESCC and poor oral health, self-reported tooth loss, and chronic periodontitis [14–16, 29–35, 37,38] (Table 1).

**Table 1.** Studies of oral health, tooth loss and periodontitis on esophageal squamous cell carcinoma.

References	Oral health status	Study design	Participant details	Relative risk (95% CI)	Adjustment
Abnet et al. [30]	Tooth loss (Median splits)	Cohort	28,868 person-cohort with 5.25 years follow-up, Linxian, China and 620 incident cases of ESCC	RR:1.3 (1.1–1.6)	Age, sex, tobacco use and alcohol use.
Wang et al. [35]	Tooth brushing (No vs. Yes)	Case–control	116 cases and 189 controls, Linfen, China	OR:0.2 (0.1–0.5)	Age, sex, residence and occupation.
Chen et al. [29]	Tooth loss (6 teeth lost vs. none)	Case–control	616 cases and 770 controls, Taixing, China	OR:1.48 (1.04–2.11)	Age, sex, education, marital status, tobacco use, alcohol consumption, family history, consumption of pickled vegetables and fresh fruits and wealth score.
	Tooth brushing (less than once vs. twice per day)			OR:1.81 (1.37–2.38)	
Hiraki et al. [37]	Tooth loss (≥ 21 remaining teeth vs. 0)	Case–control	5,240 cases and 10,480 controls, Aichi Cancer Centre, Japan.	OR:2.36 (1.17–4.75)	Age, sex, smoking and drinking status, vegetable and fruit intake, body mass index and regular exercise.
Dar et al. [31]	Teeth cleaning (≥ once vs. never per day)	Case–control	703 cases and 1,664 controls, Kashmir, India.	OR:0.44 (0.25–0.77)	Age, sex, tobacco and alcohol use, and wealth score.
Abnet et al. [32]	Decayed, missing or filled teeth (32 vs. ≤ 15)	Case–control	283 cases and 560 controls, Golestan, Iran.	OR:2.10 (1.19–3.70)	Age, sex, residence, tobacco and/or opium consumption, alcohol drinking, vegetable intake, socioeconomic status and ethnicity.
	Tooth brushing (Never vs. daily)			OR:2.37 (1.42–3.97)	
Sato et al. [34]	Tooth brushing (≥ twice vs. once per day)	Case–control	856 cases of upper aerodigestive tract cancer including 387 oesophageal cases and 2,696 controls, Aichi Cancer Centre, Japan.	OR:0.82 (0.68–0.99)	Age, sex, smoking and drinking status, vegetable and fruit intake, frequent intake of hot beverages, BMI, occupation and number of remaining teeth.
Sepehr et al. [40]	Good oral health vs. dental prosthesis	Case–control	124 cases with oesophageal dysplasia and 50 controls, Turkoman plain, Iran.	OR:4.76 (1.48–15.31)	Age, sex, ethnic origin.
Guha et al. [33]	Tooth loss (≤5 vs. 6-15)	Case–control	91 cases and 566 controls, central Europe.	OR: .84 (1.26–6.41)	Age, sex, education, country, tobacco pack-years, cumulative alcohol consumption.
			95 cases and 359 controls, Latin American	OR:2.18 (1.04–4.59)	Age, sex, education, centre, tobacco pack-years, cumulative alcohol consumption.
Abnet et al. [39]	Tooth loss (0 − 10 vs. 11-31)	Cohort	29,124 person-cohort with 14–year follow-up and 49 incident cases of ESCC.	HR:0.92 (0.46–1.83)	Age, education and H. pylori.
Nwizu et al. [14]	Self-reported periodontal disease	Cohort	65,869 women cohort with 8.32-year follow-up and 34 incident cases of oesophageal cancer	HR:3.28 (1.64–6.53)	Age, smoking status, BMI, history of diabetes and HT use.
Wen et al. [48]	Periodontitis vs. gingivitis	Cohort (Retrospective)	15,3566 participants with 13-year follow-up and 82 incident cases of oesophageal cancer.	HR:1.50 (0.97–2.31)	Age, sex and comorbidities including diabetes, hypertension, hyperlipidaemia.
Chou et al. [49]	Periodontitis (Mild vs. severe)	Cohort	25.485 individuals with mild chronic periodontitis and 25,485 individuals with severe chronic periodontitis using 1:1 propensity score matching.	HR:1.15 (0.62–2.15)	Age, sex, diabetes, cholecystectomy, Charlson comorbidity index score, medication use (aspirin and non-steroidal anti-inflammatory drugs, estimated monthly income and education level.
Michaud et al. [16]	Periodontitis (No vs. yes)	Cohort	48,375 men with median of 17.7-year follow-up and 131 incident cases of oesophageal cancer.	HR:1.44 (0.98–2.11)	Ethnic origin, BMI, physical activity, smoking history, history of diabetes, geographical region, height, alcohol, vitamin D score, calcium intake, fruit and vegetable intake, red-meat intake, and total calorific intake.
	Tooth loss (25-32 vs. 0-16 teeth remaining)			HR:1.34 (0.78-2.30)	

### Tooth loss

**Figure 1.**
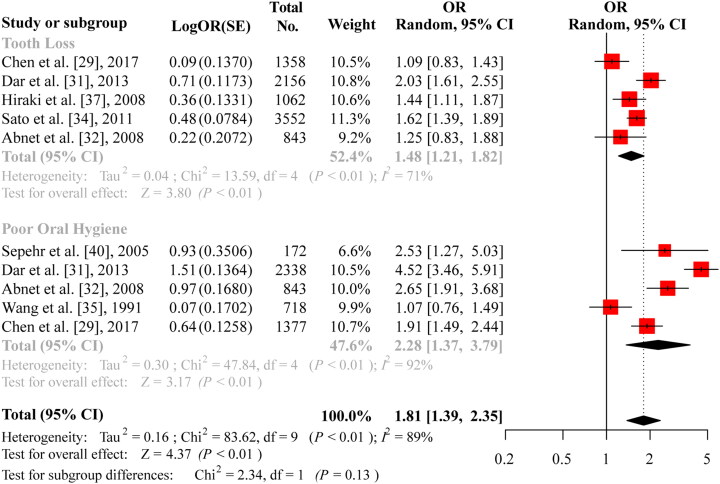
Forest Plot for the associations between oesophageal squamous cell carcinoma risk and tooth loss, and poor oral hygiene. The diamonds indicate best estimate of the true (pooled) risk with width indicating 95% confidence intervals (CI). for details, see text.

The earliest evidence suggesting an etiological relationship between ESCC and poor oral health came from a general population-based prospective study conducted in 1985 in Linxian (renamed Linzhou in 1994), located in the Taihang Mountain range in northern China, with a 100-fold higher mortality rate of oesophageal cancer than that of Caucasian Americans. In this 28,868-person cohort with a prospective follow-up of 5.25 years, tooth loss was significantly associated with cancer risk increases in ESCC, GCA, and gastric non-cardia adenocarcinoma. Furthermore, the greatest cancer risk increase was strongly correlated with the loss of the first few teeth [30] (Table 1). In line with this, another population-based case-control study conducted in Taixing, another high-incidence area for ESCC in China, also revealed that the number of tooth loss was significantly associated with ESCC risk in a stepwise manner, and that the increased risks were stronger in patients ≥ 70 years of age, women, non-smokers, and non-drinkers in the stratification analyses [29]. In Japan, one large-scale, case-control study recruiting 5,240 cancer cases and 10,480 non-cancer controls reported that tooth loss significantly increased the risk of cancers from the head and neck, oesophagus, and lung [37] (Table 1). Furthermore, a case-control study performed in Golestan Province in Iran, a high-risk region for ESCC, demonstrated that poor oral hygiene and tooth loss were significantly associated with a progressively increased risk of ESCC, with the highest risk for ESCC in subjects who lost teeth earlier [32] (Table 1). In addition, positive associations of ESCC risk with tooth loss and poor oral health have been documented in other areas as well, such as India [31], Latin America and Eastern Europe [33] (Table 1). However, inconsistent with these results, tooth loss had no effect on the risks of ESCC or oesophageal/gastric cardia adenocarcinoma, but significantly increased the risk of gastric non-cardia cancer in a prospective Finnish cohort of male smokers [39] (Table 1). The reasons for this discrepancy may be threefold: first, the contributable risk factors for these three types of cancer arising from oesophagus and stomach are distinct in these two populations, that is, Linzhou, northern China versus Finland; second, the small number of incident ESCC cases (49 cases in Finland vs. 620 cases in Linzhou) and oesophageal/gastric cardia adenocarcinoma cases (66 cases in Finland vs. 431 cases in Linzhou) compared with those of gastric non-cardia cancer cases (179 cases in Finland vs. 102 cases in Linzhou) may limit the power of statistical analysis; third, no adjustments were performed for other potential confounders linking to tooth loss or periodontitis during the statistical analysis, such as smoking, dental caries, diabetes mellitus, and socioeconomic level. To further identify the potential role of oral pathogens in ESCC tumorigenesis, a population-based screening trial was initiated to examine oral health and hygiene status in high- and low-incidence regions for ESCC in China. In agreement with the findings of previous studies in China, our group observed that tooth loss and poor oral hygiene were more common in high-incidence areas than in low-incidence areas for ESCC in China. The pooled meta-analysis also revealed that more lost teeth was significantly associated with increased OR in ESCC (OR, 1.48; 95% confidence interval (CI), 1.21 − 1.82; Figure 1). As tooth loss is mainly caused by periodontitis due to dysbiosis in the oral cavity, it is tempting to speculate that tooth loss status may reflect oral health status and is closely correlated with microbial balance in the oral cavity. In addition, an alternative potential mechanism is that tooth loss may cause incomplete chewing and rapid swallowing of hot and large pieces of food.

### Tooth brushing

As tooth brushing is closely related to oral health and hygiene, several studies have shown that tooth brushing frequency is inversely associated with ESCC risk [29–32, 34,35] (Table 1). A case-control study conducted in Linfen, Shanxi, China, revealed that brushing teeth, an indicator of oral hygiene, was strongly associated with a lower risk of EC incidence [35] (Table 1). Furthermore, a population-based case–control study performed in Taixing, China, showed that infrequent tooth brushing contributed to an 80% excess risk of ESCC [29] (Table 1). In Iran, poor oral health was associated with a higher risk of precancerous lesions and squamous dysplasia in a dose-response fashion [40] (Table 1). Similar to tooth loss, never brushing teeth or brushing teeth less than once daily was significantly associated with an increased OR (OR, 2.28; 95% CI, 1.37 − 3.79). Furthermore, our meta-analysis also reveals a significant association between ESCC risk and the combination of tooth loss and poor oral hygiene indicated by tooth brushing (OR, 1.81; 95% CI, 1.39 − 2.35; Figure 1; Supplementary file 1).

### Periodontitis

As dental caries also accounts for a proportion of tooth loss, particularly in younger individuals [41], it seems that tooth loss is not an optimal surrogate marker for periodontitis. Chronic periodontitis, characterized by chronic oral biofilm-mediated inflammation, are the major cause of tooth loss in the world and contribute to progressive destruction of the periodontium and alveolar bone loss [42, 43]. The development of periodontitis is strongly associated with oral bacteria, including *Porphyromonas gingivalis*, *Actinobacillus actinomycetemcomitans*, *Tannerella forsythensis*, and *Treponema denticola* [13, 44]. Thus, periodontitis is directly related to the microbial environment in the oral cavity. Several studies have linked periodontitis to many systemic diseases, including cancer [14, 16, 45–49]. One prospective cohort study, recruiting 65,869 postmenopausal women with a mean follow-up of 8.32 years in the USA, found that periodontal disease increased the risk of total cancer by 15% (hazard ratio (HR), 1.14; 95% CI, 1.08 − 1.20), with a stronger increased risk in the upper gastrointestinal tract, including the oesophagus and stomach (HR, 2.04; 95% CI, 1.35 − 3.09), among which oesophageal cancer risk was highest (HR, 3.28; 95% CI, 1.64 − 6.53) [14] (Table 1). A population-based retrospective cohort study in Taiwan reported that patients with periodontitis but not gingivitis exhibited a 1.14 times higher risk for total cancer, a 1.79 times higher risk for oral cancer, and a 1.5 times higher risk for oesophageal cancer compared with the control cohort [48] (Table 1). Relative to mild chronic periodontitis, the severity of chronic periodontitis did not increase the risk of total gastrointestinal cancers or individual gastrointestinal cancers, including esophageal cancer in Taiwan [49] (Table 1). However, in 48,375 older male health professionals with a median follow-up of 17.7 years in the USA, periodontitis was not associated with oesophageal cancer after adjusting for smoking status, in contrast to a 14% increased risk for overall total cancers in this male cohort [16] (Table 1). The inconsistency between males and females in the USA may stem from less smoking exposure in females, and the high prevalence of smoking in males contributes more to oesophageal cancer risk than other risk factors. Altogether, these findings indicate that a dysregulated microbiome leading to poor oral hygiene and periodontitis may play a causative role in the development of ESCC, which warrants further in-depth delineation of the composition of oral microflora and specific pathogens in relation to ESCC.

#### Oral microbiota

The oral microbiome comprises over 700 different bacterial species, representing greater than 11 bacterial phyla and 70 genera [50]. The majority of oral microbiome are commensal bacteria and play an important role in maintaining oral health, but a few species are real pathogenic factors in the development of certain oral diseases. The complex multispecies bacterial community maintains an exquisite equilibrium in the mouth ecosystem, which plays critical roles in many aspects of human health, such as immune response, carcinogen metabolism, and nutrient digestion [51–53]. Disruption of the equilibrium by *Porphyromonas gingivalis*, a key-stone oral pathogen, results in microbial dysbiosis, causing a dysregulated immune response and ultimately disease outcomes [53]. Periodontal pathogens have been identified in a number of organ systems, including lymph nodes [54], lung aspirates [55], arteries [56, 57], precancerous lesions of stomach [58] and colon [59], and oesophageal [60–63] and colorectal cancers [64–66]. These studies point to the potential implications of the oral dysbiosis in many systemic diseases, and identification of key oral pathogens would be instrumental in the development of novel preventive, diagnostic, and therapeutic strategies.

As the human oral microbiome defines all microorganisms in the oral cavity as well as its contiguous extensions up to the distal oesophagus, it is tempting to argue that oral pathogens may play crucial roles in the initiation and development of ESCC *via* local spread and dissemination stemming from the oral cavity or systemic mediators induced by a dysregulated oral microbiome. A pioneering culture-based study using oesophageal or oropharynx lavage samples isolated a common microorganism, *Streptococcus viridans*, from both the oropharynx and oesophagus. Using brush and biopsy samples from oral, upper, and lower oesophageal mucosae for both aerobic and anaerobic microbial cultivation and identification, Grusell et al. observed similar culture patterns in terms of bacterial number and diversity across samples from the oral cavity and oesophagus. In these three locations, the common bacteria were *Streptococcus viridans, Fusobacterium spp., Neisseria spp., Haemophilus spp.,* and non-pigmented *Prevotella spp.*, suggesting that the resident bacteria in oesophagus were of oral origin [67]. Using saliva from healthy subjects, a 16S rDNA-based sequencing study revealed that 43% of the clones were *Streptococcus mitis*, 13% were *S. sanguinis*, 10% were *S. parasanguis,* 7% were *S. infantis*, *S. australis*, and *S. constellatus*, 3% were *S. cristatus*, and 10% were unknown *Streptococcus* species, indicating that *Streptococcus* species are the most frequent oral commensal microbiota [60] (Table 2). In contrast, a sequencing-based study using swab samples from the uvula and biopsy samples from the proximal, middle, and distal oesophagus identified markedly different patterns of microbiome, indicating that the uvula was associated with a distinct and specific microflora as compared with all three levels of oesophagus. These data demonstrate that uvula is a distinct niche in the oral cavity and does not seem to be a surrogate of the oral cavity [68]. In a case–control study conducted in Taixing, China, which interrogated saliva microbiomes of 87 patients with ESCC, 63 cases with dysplasia, and 85 healthy subjects based on 16S rRNA gene sequencing of the V3–V4 region. The overall microbial diversity of the ESCC patients was significantly lower than that of dysplasia cases and control subjects. The most frequent phyla in the saliva included *Bacteroidetes, Firmicutes, Proteobacteria, Fusobacteria* and *Actinobacteria*. In patients with ESCC, the decreased genera included *Lautropia, Bulleidia, Catonella, Corynebacterium, Moryella, Peptococcus*, and *Cardiobacterium*, in contrast to the overabundant genera *Prevotella, Streptococcus* and *Porphyromonas*, suggesting an association between ESCC risk and aberrant oral microbiota [69] (Table 2). Chen and colleagues revealed that alpha diversity and beta diversity were distinct between the microbiota profiles of oral biofilms from healthy volunteers and ESCC patients. The abundance of *Streptococcus* species, *Prevotella* and *P. gingivalis* was significantly higher in the oral biofilms of ESCC patients than that from healthy controls. Moreover, *P. gingivalis* abundance in oral biofilm was associated with an increased ESCC risk [70]. A prospective study nested in two U.S. cohorts, that is, the NCI prostate, lung, colorectal, and ovarian (PLCO) Cancer Screening Trial Cohort and the American Cancer Society (ACS) Cancer Prevention Study II (CPS-II) Nutrition cohort, investigated the oral microbiota relationship between EAC and ESCC using 16S rRNA gene sequencing in mouthwash samples from 81/160 EAC cases, 25/50 ESCC cases, and matched controls. There were no significant associations between overall microbiota diversity or composition and risk for EAC or ESCC. Remarkably, an increased abundance of *Prevotella nanceiensis, Bergeyella oral taxon 322, Neisseria weaveri,* and *Treponema vincentii* was associated with a significantly higher risk, whereas reduced amount of *Prevotella oral taxon 306* and *Aggregatibacter paraphrophilu*s was associated with a decreased risk of ESCC. The authors also found that *P. gingivalis*, a key periodontal pathogen, was marginally associated with a higher risk of ESCC. In contrast to ESCC-related microorganisms, *Tannerella forsythia* was associated with a higher EAC risk whereas decreased abundance of the commensal genus *Neisseria* and the species *Streptococcus pneumoniae* was associated with a lower risk of EAC [71] (Table 2). It is plausible to suppose that distinct oral bacterial pathogens were linked to different subtypes of oesophageal cancer, that is, EAC and ESCC, suggesting that specific microbiota may underlie the histological origins, development, and mechanisms of EAC and ESCC.

**Table 2.** Studies of oral microbiota on oesophageal squamous cell carcinoma.

References	Study design	Participants	Sample type	Method	Results
Chen et al. [69]	Case–control	87 cases with ESCC, 63 individuals with dysplasia and 85 healthy controls from Taixing of Jiangsu, China	Saliva	(V3 to V4 regions of) 16S rRNA gene sequencing	The most common phyla in saliva included *Bacteroidetes, Firmicutes, Proteobacteria, Fusobacteria* and *Actinobacteria*; saliva microbiota in ESCC patients harboured the decreased genera including *Lautropia, Bulleidia, Catonella, Corynebacterium, Moryella, Peptococcus* and *Cardiobacterium* and the overabundant genera including *Prevotella, Streptococcus* and *Porphyromonas.*
Peters et al. [71]	Nested case–control	81 EAC cases and 160 matched controls	Mouthwash	V4 regions 16S rRNA gene sequencing	Saliva microbiota contained overabundance of *Tannerella forsythia*, and decreased abundances of the commensal genus *Neisseria* and the species *Streptococcus pneumoniae.*
		25 ESCC cases and 50 matched controls			Saliva microbiota contained overabundances of *Prevotella nanceiensis, Bergeyella oral taxon 322, Neisseria weaveri* and *Treponema vincentii,* and decreased abundances of *Prevotella oral taxon 306* and *Aggregatibacter paraphrophilu*s; *P. gingivalis* was marginally associated with a higher ESCC risk.
Narikiyo et al. [60]	Case–control	20 healthy volunteers and 58 patients with oesophageal cancer from Tokyo, Japan.	Saliva mix	16S rRNA gene amplification and clone sequencing	*Streptococcus (S.) mitis* (43%), *S. sanguinis* (13%), *S. parasanguis* (10%), *S. infantis, S. australis, S. constellatus* (7%), *S. ctirtatus* (3%) and unknown Streptococcus species (10%).

In the light of the anatomical site adjacency and high consistency in microbiota between the oral cavity and oesophagus, the oral cavity may represent a microbial window mirroring alterations in the composition and structure of the microbial ecosystem colonizing the oesophagus. Thus, the identification of key oral pathogens associated with ESCC, particularly in high-risk regions of ESCC, is a potential microbial biomarker for screening and surveillance in high-risk population, early detection, and potential targets for preventive intervention.

#### Oesophageal microbiota

The oesophagus, a luminal organ that links the oral cavity with the stomach, allows the transient passage of food into the stomach, in contrast to mouth and other parts of the gastrointestinal tract, which retain and digest food to various degrees. The luminal surface of the oesophagus is lined with ample mucosa for bacterial colonization, and an oesophageal biofilm has been described by several research groups [67, 72–75]. Under normal physiological conditions, microorganisms are introduced into the oesophagus from the mouth by swallowing or from the stomach by reflux [72]. This may support the causal relationship between esophageal diseases and pathogenic agents from the oral cavity or stomach.

**Figure 2.**
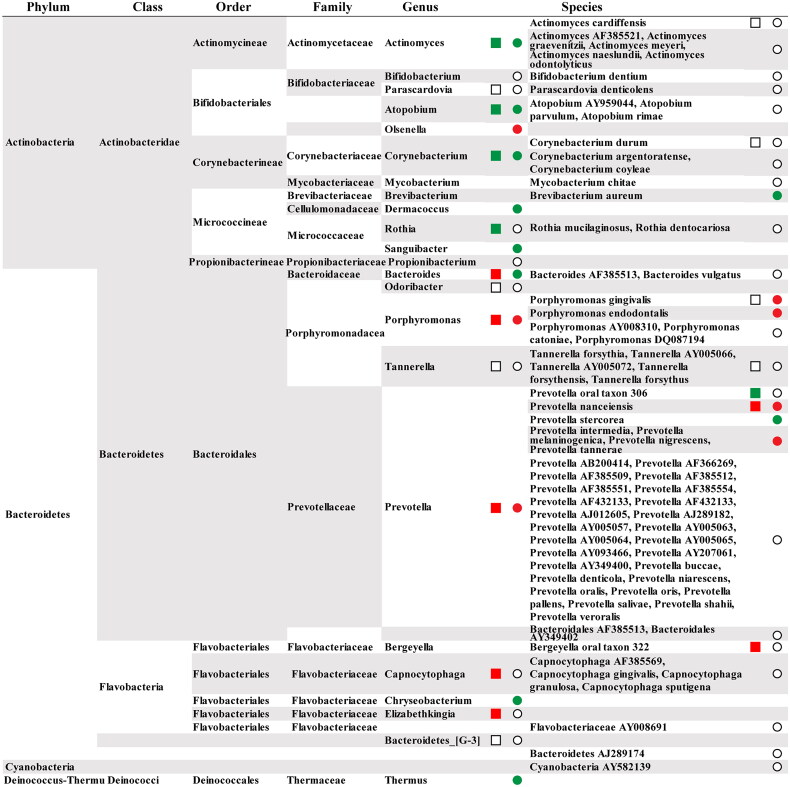

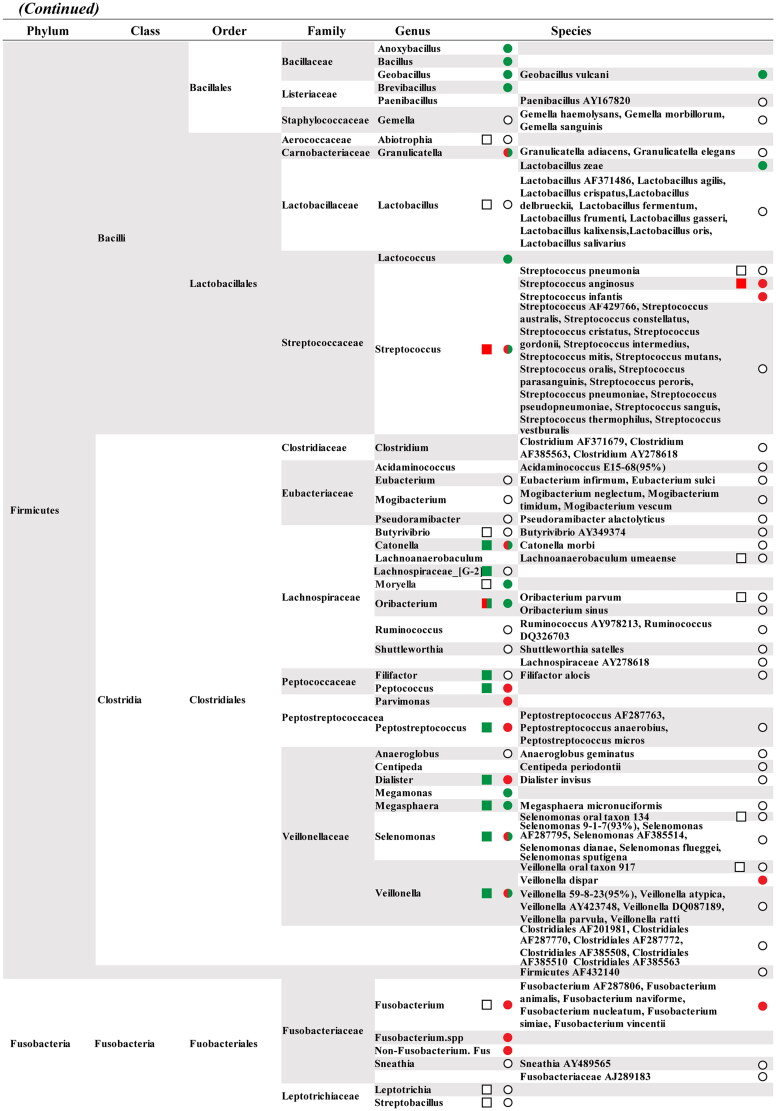

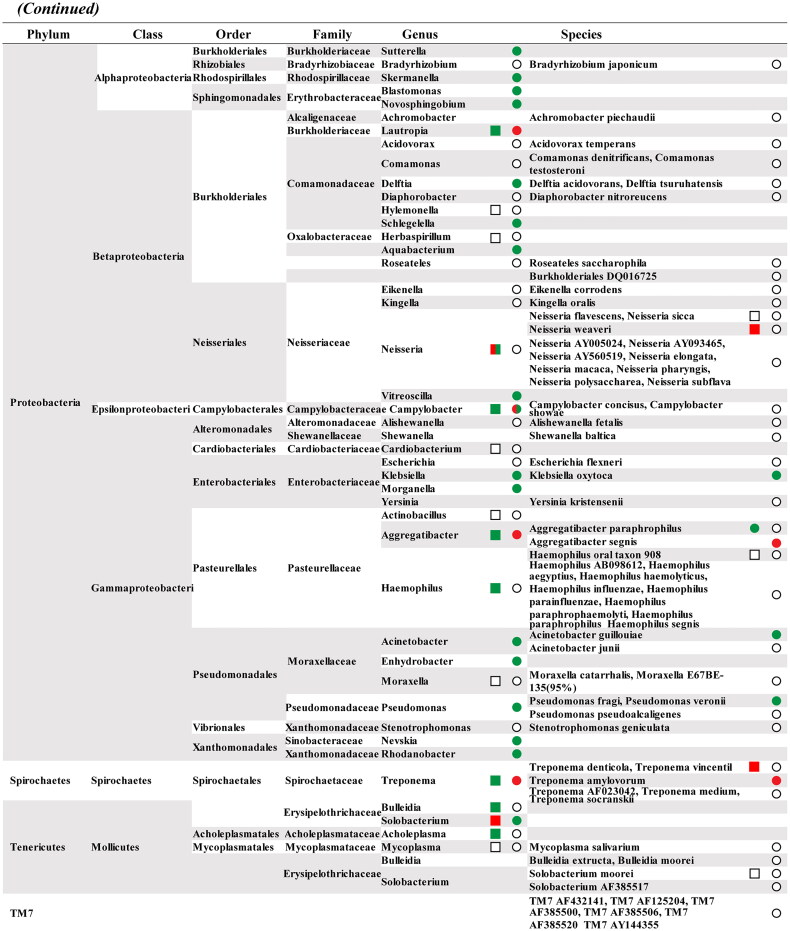
The distribution of previously published bacterial taxa derived from oral cavity and oesophagus that represented by the square and circle, respectively. The red/green indicates a detrimental and preventive bacterial taxa. For details, see text.

Using biopsy specimens from a healthy distal oesophagus, 900 16S rDNA PCR clones were identified, indicating abundant microbiota in the normal distal oesophagus. Six phyla, including *Firmicutes, Bacteroidetes, Actinobacteria, Proteobacteria, Fusobacteria*, and TM7, were found in the oesophageal microbiota. Among the 41 genus-level taxonomic units, *Streptococcus* (39%), *Prevotell*a (17%), and *Veilonella* (14%) were the most common genera. Most species-level operational taxonomic units (SLOU) of the microbiota in the distal oesophagus were similar to the up-stream oral flora [73] (Table 3). Consistent with these findings, a culture-based study found streptococcal species are the most common organisms in oesophagus [67]. Most of oesophageal bacteria (82%) are cultivable and derived from known families [73], while 70% of oral bacteria, including 57% officially named and 13% unnamed, are cultivable [76]. Accumulating studies reveal that the core microbiota in healthy oesophageal mucosa comprises *Streptococcus, Fusobacterium, Veillonella* and *Prevotella* genera, whose composition changes under pathological conditions [50, 76]. A total of 10 phyla have been identified in oral cavity and oesophagus, among which the most common phyla include *Firmicutes*, *Proteobacteria*, *Bacteroidetes* and *Actinobacteria* (Figure 2). Certainly, the normal distal oesophagus contains a complicated but conserved bacterial community that is closely related to the oral microbiota.

**Table 3.** Studies of oesophageal microbiota on oesophageal squamous cell carcinoma.

References	Participants	Sample type	Method	Results
Pei et al. [73]	Four persons with gastrointestinal symptoms	Four distal oesophageal biopsies with normal oesophageal histology	16S rDNA PCR	Six phyla, 41 genera including 13 common genera among 4 persons, and 95 SLOTU including 14 common SLOTU among 4 persons.
Yang et al. [77]	Twelve normal oesophagus, 12 oesophagitis, 10 Barrett’s oesophagus	34 biopsy samples of distal oesophagus.	16S rRNA gene amplification and clone sequencing	Nine phyla, 70 genera and 166 SLOTU; Type I microbiome dominated by *Streptococcus* in normal oesophagus; Type II microbiome represented by Gram-negative anaerobes in esophagitis and Barrett’s oesophagus
Yu et al. [78]	333 subjects including 142 with ESD and 191 without ESD from Linxian (Linzhou), Henan, China	Upper digestive tract cell samples collected by inflatable rubber balloon covered with cotton mesh or Cytomesh Oesophageal Cytology Device	Human Oral Microbe Identification Microarray for detection of 272 bacterial species.	An inverse association between microbial richness and ESD, and a positive association between microbial richness and PGI/II ratio.
Yang et al.[79]	18 patients with ESCC and 11 volunteers with physiological normal oesophagus from Guangzhou, China	18 ESCC samples and 11 biopsy samples with normal oesophagus	PCR amplification of V4 region of 16S rDNA and sequencing	ESCC tissues had significantly decreased microbial diversity and high abundances of *Bacteroidetes*, *Fusobacteria* and *Spirochaetes*, which were associated with reduced nitrate reductase and nitrite reductase function
Shao et al. [81]	67 patients with ESCC and from Linzhou, Henan, China	Paired ESCC tissue and non-tumour tissue samples	PCR amplification of V4region of 16S rDNA and sequencing	Microbial composition of ESCC comprised of *Firmicutes*, *Bacteroidetes* and *Proteobacteria* phyla; ESCC contained higher abundance of *Fusobacteria* phylum including *Fusobacterium* genus, and less abundance of *Firmicutes* phylum including *Streptococcus* genus in comparison with non-tumour tissues; ESCC harboured greater abundances of *Fusobacteria* and *Tenericutes* phyla, greater abundances of *Mycoplasma* and *Fusobacterium* genera in comparison with GCA tumour tissues.
Narikiyo et al. [60]	58 patients with oesophageal cancer from Tokyo, Japan, 4 from China, 2 from France, 5 from Italy.	Oesophageal cancer tissues with or without paired normal tissues.	16S rRNA gene amplification and clone sequencing	Frequent infection of the oral periodontopathic spirochete *Treponema denticola, Streptococcus mitis, Streptococcus anginosus* in ESCC from different regions of the world.
Yamamura, et al. [62]	325 patients with oesophageal cancer from Kumamoto, Japan	Formalin-fixed, paraffin-embedded (FFPE) oesophageal cancer tissues including 300 ESCCs, 12 adenocarcinomas and 13 of others	qPCR quantification of *Fusobacterium nucleatum*	*F. nucleatum* positivity in tumour is 23% (74/325) and is positively correlated with tumour stage. *F. nucleatum*-positive patients showed poor prognosis with multivariate HR: 1.98 (1.14-3.37)
Yamamura, et al. [63]	551 cases with ESCC	A training cohort of 207 cases with fresh frozen tissues and a validation cohort of 344 cases with FFPE tissues from Nagoya, Kumamoto, Japan, respectively.	qPCR quantification of *Fusobacterium nucleatum*	High levels of *F. nucleatum* in ESCC associate with an invasion depth, poor recurrence-free survival, poor chemotherapeutic response
Gao et al. [61]	100 patients with ESCC	100 ESCCs with paired nontumor tissues, and 30 biopsy samples or normal oesophagus from Henan,	Immunohistochemistry and qPCR of 16S rDNA	*Porphyromonas gingivalis* positivity was 61% in ESCC, 12% in adjacent tissues, and negative in normal oesophagus. High burden of *P. gingivalis* in ESCC was
		China		positively correlated with poor differentiation, lymph node metastasis, advanced TNM stage, and poor overall survival.
Gao et al. [90]	Patients with ESCC or esophagitis, and healthy controls.	96 cases with ESCC, 50 cases with esophagitis, and 80 healthy controls.	ELISA to measure the serum IgG and IgA antibodies against *P. gingivalis.*	Serum levels of IgG and IgA against *P. gingivalis* were higher in ESCC patients than those in patients with esophagitis and healthy controls. Higher levels of IgG and IgA were associated with poor prognosis.

Note: SLOTU denotes species-level operational taxonomic units.

In the human distal oesophagus, the microbiota in histologically defined normal, oesophagitis, and Barrett’s oesophagus were classified as type I or II. The type I microbiome was well represented by the *Streptococcus* genus in the phenotypically normal oesophagus, whereas the type II microbiome was dominated by Gram-negative anaerobes/microaerophiles in oesophagitis and Barrett’s oesophagus. This study identified a total of nine phyla represented by 166 species, which were associated with histological phenotypes of the distal oesophagus [77] (Table 3, Figure 2). Notably, an inverse association between microbial richness and oesophageal squamous dysplasia was observed, in contrast to the opposite in normal upper digestive tract [78] (Table 3). These results suggest that lower microbial richness in the upper digestive tract in subjects from Linxian, China, could increase the risk of cancer-predisposing states in the oesophagus and stomach. Furthermore, the ESCC tissues had significantly decreased microbial diversity and high abundances of *Bacteroidetes*, *Fusobacteria* and *Spirochaetes*, which were associated with reduced nitrate reductase and nitrite reductase function [79]. Interestingly, a recent study from the United Kingdom also reported that the abundance of genera decreased in oesophageal adenocarcinoma comprised of Gram-negative (*Veillonella, Megasphaera,* and *Campylobacter*) and Gram-positive taxa (*Granulicatella, Atopobium, Actinomyces,* and *Solobacterium*) [75]. As the prevalence of GCA concurs with that of ESCC in the Asian Oesophageal Cancer Belt [80], Dantong and colleagues characterized the bacterial microbiota of ESCC and GCA with paired nontumor tissues from Linzhou, China. The microbial composition of ESCC and GCA tissues was primarily composed of *Firmicutes*, *Bacteroidetes* and *Proteobacteria* at the phylum level. ESCC harboured a greater abundance of *Fusobacterium* (3.2% vs. 1.3%) and a lower abundance of multiple taxa such as *Streptococcus* (12.0% vs. 30.2%) than nontumor tissues, whereas GCA contained a higher abundance of multiple taxa, including *Firmicutes, Bacteroidetes, Fusobacteria, Actinobacteria* and *Proteobacteria* phyla, and *Helicobacter* genus (60.5% vs. 11.8%) than nontumor tissues. Significant clustering between paired ESCC/GCA and nontumor tissues was found based on β-diversity, reflecting the microbial community composition by the weighted UniFrac distance [81] (Table 3). These results demonstrate that the microbial ecosystems of ESCC and GCA are distinct despite anatomical site adjacency, which warrants further validation. The abundance of *P. gingivalis* and *T. forsythus* was significantly enriched in ESCC compared with adjacent nontumor and healthy control tissues using 16S rDNA sequencing of the oesophageal mucosa biopsy of healthy controls, ESCC, and matched adjacent nontumor tissues. Notably, we also found that *P. gingivalis*-associated risk for ESCC was predominantly observed in ESCC patients with early clinical stage, and enrichment of *Ruminococcus* genus was positively correlated with poor prognosis in Anyang, a high-incidence region for ESCC. In contrast, using cloning and Sanger sequencing of 100 clones, Michihiro and colleagues reported frequent infection of the oral periodontopathic spirochete *Treponema denticola, Streptococcus mitis, Streptococcus anginosus* in ESCC, all of which are of oral origin and present in nearby non-tumour tissues. The oesophageal cancer samples used in this study were from Japan, China, France, and Italy, without histologic subtype information [60] (Table 3). The reasons for the discrepancy between these two studies may include different detection methods and tumour histology.

*Fusobacterium nucleatum*, a proportion of the normal flora inhabiting the human oral cavity, vagina, and gut, is an opportunistic pathogen implicated in a variety of inflammatory diseases and contributes to the development of many human cancers [59, 62, 65, 66, 82, 83]. The abundance of *F. nucleatum* was significantly increased in ESCC than in matched normal oesophageal mucosa and was positively associated with advanced tumour stage [62] (Table 3). Additionally, ESCC patients with higher abundance of *F. nucleatum* had significantly shorter cancer-specific and overall survival than those with lower *F. nucleatum* abundance. Furthermore, a higher abundance of intratumoral *F. nucleatum* in ESCC patients was correlated with a worse response to neoadjuvant chemotherapy and higher tumour recurrence [63] (Table 3). As a keystone pathogen in chronic periodontitis [84, 85], *P. gingivalis* was present in many extraoral infection-related diseases, such as cardiovascular diseases, diabetes, rheumatoid arthritis, Alzheimer’s disease, and various cancers [86–88]. A meta-analysis revealed that the prevalence of *P. gingivalis* in cancer patients was significantly greater than that in healthy controls, and *P. gingivalis* carriers have a 1.36-fold increase in risk for cancer and periodontitis [89]. Colonization of *P. gingivalis* in oesophageal mucosa was detected and the prevalence of *P. gingivalis* in ESCC was 61% compared with 12% in matched adjacent nontumor tissues. A high abundance of *P. gingivalis* in ESCC was positively associated with dismal survival and aggressive clinical features, including poor differentiation, lymph node metastasis, and advanced clinical stage [61] (Table 3). Furthermore, the median serum levels of IgG and IgA antibodies against *P. gingivalis* increased remarkably in patients with ESCC compared to those in non-ESCC controls. The combination of IgG and IgA against *P. gingivalis* produced a sensitivity and specificity of 68.75% and 68.46%, respectively, for the diagnosis of ESCC and even performed well in early ESCC. Furthermore, higher levels of IgG and IgA antibodies against *P. gingivalis* predicted worse prognosis in ESCC patients [90] (Table 3). These results indicate that *F. nucleatum* and *P. gingivalis* may play causative and tumorigenic roles in the onset and development of ESCC, which requires further investigation. Furthermore, tumor enrichment of *F. nucleatum* and *P. gingivalis* represents potential prognostic and predictive biomarkers for the clinical management of ESCC and potential targets of antibiotic intervention for improving the therapeutic response in ESCC patients.

#### Gastric microbiota

Despite being hostile for bacterial survival in the normal human stomach, 16S rRNA gene sequencing identified a very complex bacterial flora inhabiting the acidic niche of the stomach (Table 4), which refutes the dogma that the stomach is a sterile organ [24, 92]. *Helicobacter pylori* infects half of the world population [91] and is the most abundant bacterium in the gastric microbiota of *H. pylori*-positive subjects. The presence of *H. pylori*, however, did not affect the diversity and composition of gastric bacterial community [93]. Apart from *H. pylori*, the gastric microbial community is distinct from the oral, oesophageal and respiratory microflora despite the majority of bacteria reminiscent of upstream components of alimentary and respiratory tracts [94]. The core gastric microbiota comprises five major phyla, that is, *Proteobacteria*, *Firmicutes, Actinobacteria, Bacteroidetes* and *Fusobacteria* [93], common to those of oesophageal microbiota.

**Table 4.** Studies of gastric microbiota on esophageal squamous cell carcinoma.

References	Participants	Sample type	Method	Results
Bik et al. [93]	23 adult subjects comprising 12 *H. pylori*-positive and 11 *H. pylori*-negative subjects	9 gastric corpus biopsy samples and 14 gastric antrum biopsy samples	16S rRNA gene amplification and clone sequencing	A total of 128 phylotypes were assigned to five major phyla, i.e. *Proteobacteria*, *Firmicutes, Actinobacteria, Bacteroidetes* and *Fusobacteria*; no significant association between gastric microbial composition and *H. pylori* status, gastric anatomical location, and gastric pH value; gastric microbiota is distinct from oral and oesophageal microbiotas.
Nasrollahzadeh et al. [95]	Case cohort comprising 19 patients with stage I to II ESCC and 18 patients with oesophageal squamous dysplasia (ESD), 17 subjects with esophagitis as disease cohort, and 37 normal oesophagus as healthy control cohort from Golestan, Iran	Snap frozen gastric corpus tissue samples	PCR amplification of V3-V4 region of 16S rDNA and sequencing	Five major core phyla across ESCC, ESD, esophagitis and normal oesophagus; higher abundances of *Clostridiales* and *Erysipelotrichales* orders in patients with ESCC or ESD; no microbial composition difference between esophagitis and normal oesophagus.
Shao et al. [81]	36 patients with GCA from Linzhou, Henan, China.	Paired GCA tissue and nontumor tissue samples	PCR amplification of V4 region of 16S rDNA and sequencing	Microbial composition of GCA comprised of *Firmicutes*, *Bacteroidetes* and *Proteobacteria* phyla; GCA contained higher levels of *Firmicutes*, *Bacteroidetes*, *Fusobacteria*, *Actinobacteria* phyla; higher levels of *Prevotella, Streptococcus, Veillonella, Haemophilus* and *Neisseria* genera, and lower levels of *Proteobacteria* phylum and
				*Helicobacter* genus in comparison with nontumor tissues; GCA had higher abundance of *Proteobacteria* phylum and *Helicobacter* genus in comparison with ESCC.
Yu et al. [96]	80 gastric cardia cancer patients from Taiyuan, Shanxi, China	Paired GCA tissue and nontumor tissue samples	PCR amplification of V3-V4 region of 16S rDNA and sequencing	GCA had higher amounts of *Bacteriodetes*, *Firmicutes, Fusobacteria* and *Spirochaetes* phyla, lower amounts of *Proteobacteria* phylum, *Helicobacter* genus and *H. pylori* compared to non-malignant tissues; either *H. pylori* status or gastric anatomic sites had no effects on gastric microbiota composition;
Yu et al. [97]	80 gastric cardia cancer patients from Taiyuan, Shanxi, China	Paired GCA tissue and non-tumour tissue samples	PCR amplification of V3-V4 region of 16S rDNA and sequencing	In non-malignant tissue, higher level of *H. pylori* was positively associated with the family history of upper gastrointestinal cancer and the tumour grade; higher abundance of *Lactobacillales* in patients without metastasis; higher abundance of *Bacteroidetes* in patients with lower tumour grade.

Chronic atrophic gastritis caused by chronic *H. pylori* infection is associated with an increased risk of ESCC as well as its precursor, oesophageal squamous dysplasia [38, 78]. Nevertheless, the tumorigenic effect of *H. pylori* infection may be more potent for gastric cardia mucosa than for oesophageal squamous mucosa. In a study conducted in Golestan, Northern Iran within the ‘oesophageal cancer belt’, gastric corpus microbiota harboured higher relative abundance of orders *Clostridiales* and *Erysipelotrichales* in cases with early ESCC and ESD compared with those of diseased and healthy controls. Although accounting for nearly 43% of the total reads, *Helicobacteriacea* failed to cluster the microbial communities in terms of the pathological status of the oesophagus. Based on the Unifrac distance and weighted Unifrac distance, the pattern of the gastric microbiota was significantly altered between cases and controls [95] (Table 4). Although co-occurrence of ESCC and GCA in the Asian ‘oesophageal cancer belt’ including Linzhou, China, the microbial compositions in these two types of cancer with distinct histology were different. The relative amounts of genera *Helicobacter* and *Proteobacteria* were higher in GCA, whereas ESCC was enriched with higher levels of genera *Mycoplasma* and *Fusobacterium*, and phyla *Fusobacteria* and *Tenericutes*, and less *Streptococcus*. In comparison with matched non-tumour tissue, GCA showed a decreased relative abundance of *helicobacter* genus [81] (Table 4). In Taiyuan, Shanxi, which has a high incidence of GCA, 16S rRNA gene sequencing analysis of GCA revealed that the microbiota of GCA had lower amounts of *Proteobacteria* and greater amounts of *Bacteriodetes*, *Firmicutes, Fusobacteria* and *Spirochaetes* than non-malignant tissues. The relative abundances of the genera *Helicobacter* and *H. pylori* in GCA were lower than those in matched non-malignant gastric tissues. Although *H. pylori* colonization was present in 94% of the gastric tissue of patients with GCA, the gastric microbiota composition did not differ according to *H. pylori* infection or sub-anatomic sites in the stomach [96] (Table 4). Nevertheless, no associations between the features of the GCA microbiota and clinical variables were uncovered. In contrast, the microbiota features in matched nontumor tissues were correlated with a family history of upper gastrointestinal tract cancer, tumour grade, and metastasis. A higher relative abundance of *H. pylori* was detected in patients with a positive family history and an advanced tumour grade. Overabundance of *Bacteroidetes* was associated with lower tumour grade, and higher abundance of *Lactobacillales* was negatively associated with metastases, suggesting a protective role for these two microoranisms [97] (Table 4). These results demonstrate that distinct microbial communities exist in non-malignant and tumor tissues in ESCC and GCA, which underlies the potential for the identification of distinguishable preventive, diagnostic, and therapeutic microbial targets between ESCC and GCA.

#### Virus

The complex microbial community of the microbiota includes bacteria, archaea, fungi, protozoa, and viruses. Apart from certain cancers related to bacterial infection, viruses cause approximately 10% of all human cancers worldwide [98]. Several studies have demonstrated that infection with oncogenic viruses may play potential direct pathogenic roles in the development of ESCC.

### Human papilloma virus (HPV)

The role of HPV in ESCC development and progression remains controversial. The pioneering study reporting the close relationship between HPV and ESCC dates back to 1982 by Kari Syrjänen, who subsequently published several updated literature reviews summarizing this topic in 2002, 2006, 2010, and 2013 [99, 100]. The prevalence of HPV DNA in ESCC tissues is characterized by great variation with a range of 0% to 70% [101]. To date, the latest systematic review involving the largest number of ESCC cases included 14 788 cases from 187 studies performed in 32 countries from six continents (Africa, Asia, Australia, Europe, North America, and Latin America) published between 1982 and 2017 [102]. In agreement with previously published studies [100, 101, 103–108], the overall prevalence of HPV among these 14 788 cases was 30.9% (95% CI, 30.1–31.6%) with remarkable geographical differences. The highest prevalence of HPV was from China (40.0%; 95% CI, 39.0–41.1%), followed by South Africa (29.7%; 95% CI, 26.0–33.6%), and several other Asian countries, with the exception of China, which is among the highest incidence area of ESCC. In addition, forty-two reports from 12 countries between 2012 and 2017 revealed that *Alpha-PV* prevalence of alpha-PV was 31.1% (95% CI, 29.8-32.4%), of which HPV16/18 prevalence was 73.8% (95% CI, 71.5–76.0%). The most common HPV type detected in ESCC is HPV 16 followed by HPV 18. Integration of HPV16 into the host genome contributes to malignant conversion and cancer in the oesophageal epithelia [109].

In sharp contrast, only 1.1% of HPV infections were detected in 272 cases of ESCC from China, including Linxian, contradicting the etiological association between HPV and ESCC [110]. A prospective nested case-control study reported that the prevalence of alpha, beta, and gamma HPV in the oral cavity did not increase significantly in incident oesophageal cancer cases compared to controls matched for age, sex, and race/ethnicity. Moreover, none of 28 ESCC cases showed positive oral HPV 16 [111]. Using centralized multiplex serological assays to detect antibodies against L1 and E6/E7, only four cases and two controls were positive for HPV E6 and E7 among 1561 case subjects and 2502 controls subjects, respectively, from six case–control studies [112]. In 86 patients with early ESCC undergoing endoscopic resection or esophagectomy in France, RNAscope HPV-HR18 Probe was performed to detect 18 high-risk HPV genotypes and no cases were positive for the HPV types tested [113]. Furthermore, there was no evidence of integrated HPV DNA in ESCC samples from Western and Eastern countries by genome sequencing analysis [114, 115]. These findings indicate that HPV plays a negligible role in the pathogenesis of ESCC.

### Epstein–Barr virus (EBV)

EBV is a member of the gamma-herpes virus family and preferentially infects B-lymphocytes as well as epithelial cells. It is a widespread oncovirus that affects more than 95% of all adults as a lifelong asymptomatic latent infection [116, 117]. Several studies have indicated associations between EBV and a variety of malignancies, such as multiple types of lymphoma, nasopharyngeal carcinoma, and gastric adenocarcinoma. EBV infection contributes to approximately 2% of all cancer-related mortality per year [118, 119]. Since the first report revealed the presence of EBV in microdissected ESCC samples in 1994, the frequency of EBV positivity ranged from 0% to 35.5% in ESCC tissues [104, 120, 121]. The whole-exome sequencing of 90 ESCC samples by The Cancer Genome Atlas (TCGA) failed to find evidence of EBV infection [114]. In line with this, none of the esophageal cancer samples in a large cohort of 988 esophageal cancer samples were EBV-encoded RNA (EBER) positive by EBER *in situ* hybridization [121]. The conflicting results on the association between EBV and ESCC can be attributed to different detection techniques and geographic factors. However, the very rare ESCC variant of lymphoepithelioma-like carcinoma is frequently EBV-positive and results in an improved outcome compared with conventional ESCC, probably due to hypermutation induced by EBV infection in parallel with heavy lymphocyte infiltration [118]. Nevertheless, despite the general null relationship between EBV and ESCC, at least some ESCC subtypes exhibit evidence of EBV infection in ESCC, to some extent, in certain races and countries.

### Other viruses

In addition to the extensively studied viruses such as HPV and EBV, other viruses may play an etiological role in the development of ESCC in a similar fashion. These viruses include herpes simplex virus (HSV), cytomegalovirus (CMV) [122], Merkel cell polyomavirus (MCPyV) [104, 123] and John Cunningham virus (JCV) [124], which could serve as cofactors for HPV-related carcinogenesis.

In the oesophagus, HSV causes esophagitis as a distinct clinical entity. To date, three studies have been performed to determine the prevalence of HSV in ESCC. Among these three studies, two studies used ESCC samples from the high-incidence population of Shantou China and the HSV prevalence was 31.7% and 30.0%, respectively [125, 126]. In sharp contrast, another study investigated HSV infection by immunohistochemistry in 103 ESCC samples from Anyang, northern China, and none of the tested samples were HSV-positive [122]. The discrepancy of HSV infection in ESCC may be associated with geographic variation.

Although CMV, MCPyV, and JCV are well-recognized causative agents of other malignancies, few studies have reported their prevalence in ESCC tissues with variation. Thus, conclusive evidence of the etiological role of these viruses in ESCC is still lacking, and more studies are warranted.

#### Potential mechanisms of microbiota-associated tumorigenesis in ESCC

Accumulating data have demonstrated that the cancer microbiome plays a key role in the aetiology and development of some types of cancer including ESCC [127]. Elucidating the cancer-promoting mechanisms holds the potential for rational development of microbiome-based detection, prevention and treatment. Nowadays, multiple clinical and experimental studies have revealed the mechanisms which enhance malignant progression through microbial entity, cancer cell, tumour microenvironment, and immune escape.

The first potential mechanism is inflammation which has been recognized as a hallmark and cause of cancer [128]. *P. gingivalis* and *F. nucleatum*, two potential pathogens with oral origin, can induce chronic inflammatory response and establish a dysbiotic milieu which disrupts the local immunoinflammatory equilibrium [129, 130]. *P. gingivalis* infection led to upregulation of IL-6 and TGFβ1 in cultured ESCC cells and in ESCC specimens [131, 132]. In *F. nucleatum*-infected ESCC tissues, cytokine-cytokine receptor interaction was the significant enriched pathway and CCL20 was the most upregulated chemokine [62]. Second, the inflammatory cells in tumour environment can promote cancer cell proliferation as well as suppress antitumor immunity. Accumulation of CD11b^+^ myeloid cells in inflammatory microenvironment was observed in orthotopic MC38 rectal cancer tissues [133]. *P. gingivalis* can induce upregulation of B7-H1, B7-DE, and B7- H4, which inhibit effector T cells and cause immune evasion [130, 134]. Third, growing evidence suggests that intratumor microbiome and gut microbiome cancer can significantly influence response to cancer therapy [135, 136]. Microbiome ablation by oral antibiotic administration prevents oncogenic progression. Both *P. gingivalis* and *F. nucleatum* in ESCC are associated with worse chemotherapeutic efficacy and have the potential to predict response to neoadjuvant chemotherapy in patients with ESCC [63, 137]. Fourth, microbial metabolites from dietary and environmental sources contributes to the initiation and development of ESCC [2]. Dysbiosis of esophageal microbiota led to dysregulation of nitrate reductase activity [79]. Nitrosamine was increased in subjects with poor oral hygiene and the increase of nitrosamine was significantly reduced by antiseptic mouthwashes [30]. Acetaldehyde, another carcinogen in upper gastrointestinal tract, is produced from ethanol by oral bacteria, such as *P. gingivalis* [*2*]. Lastly, microbial entity itself produce a variety of virulence factors, such as LPS, gingipains, and FimA, Fap2 and FadA adhesins, which play a role in initiation and development of malignancies [61, 128, 130]. Through interaction with cancer cells, pathogenic bacteria in upper gastrointestinal tract promotes survival and suppresses death *via* subversion of multiple signaling pathways, including PI3K/Akt, ERK, TGFβ, Jak1/Stat3, and miR194/GRHL3/PTEN pathways [128, 130, 132, 138].

#### Conclusions and future challenges

Lifestyle factors, including dietary habits, oral healthcare, tobacco smoking, and alcohol consumption, play critical roles in the aetiology of ESCC. Many epidemiological studies have provided supportive evidence for poor oral hygiene status, such as tooth loss and chronic periodontitis, as risk indicators for ESCC. With the growing appreciation of microbiota in the pathophysiology of multiple diseases, including ESCC, beneficial and detrimental bacterial candidates implicated in aggressive progression and impact on therapeutic response and recurrence of ESCC have been increasingly identified. However, the causality of these detrimental bacteria, such as *P. gingivalis* and *F. nucleatum*, in the initiation and development of ESCC warrants experimental evidence and follow-up of high-risk subjects with precancerous lesions. Additionally, the etiological role of oncogenic viruses in the initiation and development of ESCC remains unclear. Further in-depth studies are needed to elucidate the biological mechanisms underlying the suppressive or promoting roles of these distinct microbial patterns in ESCC pathogenesis. The next challenge is to translate dysbiotic microbiota and related signaling effectors and metabolites into clinical utility as potential biomarkers for diagnosis, prognostication, and therapeutic targets [127].[AQ]

## Supplementary Material

Supplemental Material

## Data Availability

All data generated or analysed during this study are included in this published article.
