# Supplementary material for: Upper gastrointestinal tract microbiota with oral origin in relation to oesophageal squamous cell carcinoma
Source: Ann Med. 2023 Dec 27;55(2):2295401. doi: 10.1080/07853890.2023.2295401 (PMC10763922; doi:10.1080/07853890.2023.2295401)

Supplementary files 1

Protocol for Meta-analysis of oral health as in relation to the risk of ESCC.

We conducted a literature search from PubMed, Web of Science, and The Cochrane Library for relevant articles published before December 31, 2022. Taking PubMed as an example, the following search keywords were used: (1) Oral care, Oral health, Oral hygiene, Dental health, Dental hygiene, Tooth loss, Teeth loss, Tooth absence, Missing teeth, Edentulism, Toothbrushing, Tooth brushing, Teeth brushing, Mouthwash, Mouthwashes, Oral hygiene practices, Tooth cleaning. (2) Esophageal, Esophagus, Oesophagus, Oesophageal. (3) Cancer, Carcinoma, Tumor, Neoplasm. The search was performed by combining the terms using the "and" operator for (1), (2), and (3), resulting in a total of 1549 relevant articles being identified.

To evaluate the power of association between tooth loss and ESCC, poor oral hygiene and ESCC, pooled measure was calculated as the inverse variance-weighted mean of the logarithm of effects (ORs with 95% CI). The *Q* test and Higgins *I^2^* statistics was used to assess the among-study heterogeneity. The Dersimonian and Laird random effects model (REM) was used as the pooling method because the heterogenities of the articles involved in the present study are > 50%. Data analyses were carried out using R software meta package. All reported probabilities (*P* values) were two-sided and considered significant if *P* values less than 0.05.

Figure S1 Flow diagram of the search for articles.


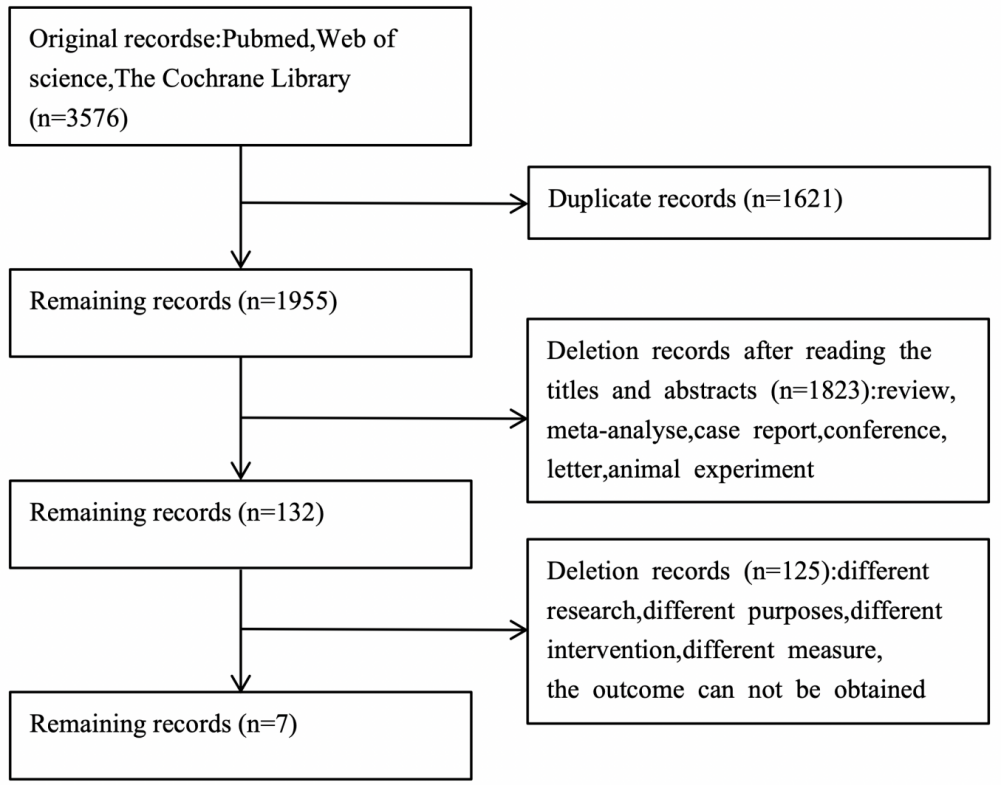

Supplement: Supplemental Material [file IANN_A_2295401_SM3749.docx]
